# Men’s Healing Work: Reconciling Intergenerational Ambivalence Through Fatherhood, Letter Writing, and Community

**DOI:** 10.1093/geroni/igaa057.1115

**Published:** 2020-12-16

**Authors:** Allen Kim

**Affiliations:** International Christian University, Tokyo, Japan

## Abstract

Due to globalization, new ideals of fatherhood are challenging traditional paternal roles in South Korea. Contemporary fathers striving to emulate more engaged parenting sometimes wrestle with painful recollections of their own fathers’ stern, distant, and patriarchal approach. How do men reconcile their aspirations for their own development as parents when conflicted relationships with their fathers? Motivated by the concept of intergenerational ambivalence, this study analyzes letters South Korean men write to their fathers as assigned homework for Father School, an international men’s movement that aims to make men more nurturing. Under Father School direction, men adopt a life course frame that allows them to reconcile their mixed feelings toward their aging fathers. Analysis points to three life course discursive strategies that permit men to balance negative judgments with positive ones: 1) sharing with their parent the life stage as worker and father; 2) appreciating historically-situated differences between twentieth and twenty-first century lives and parenting imperatives; and, 3) drawing on deeply rooted filial norms to take responsibility for their own role in intergenerational conflicts. In addressing how these men manage intergenerational ambivalence, the article moves beyond prior research to extend the concept to father-son dyads, the Asian context, and the neglected meso-level where organizations may actively structure reconciliation.

